# Tying up the Loose Ends: A Mathematically Knotted Protein

**DOI:** 10.3389/fchem.2021.663241

**Published:** 2021-05-24

**Authors:** Shang-Te Danny Hsu, Yun-Tzai Cloud Lee, Kornelia M. Mikula, Sofia M. Backlund, Igor Tascón, Adrian Goldman, Hideo Iwaï

**Affiliations:** ^1^ Institute of Biological Chemistry, Academia Sinica, Taipei, Taiwan; ^2^ Institute of Biochemical Sciences, National Taiwan University, Taipei, Taiwan; ^3^ Institute of Biotechnology, University of Helsinki, Helsinki, Finland; ^4^ Division of Biochemistry, Department of Biosciences, University of Helsinki, Helsinki, Finland; ^5^ Astbury Centre for Structural Molecular Biology, School of Biomedical Sciences, University of Leeds, West Yorkshire, United Kingdom

**Keywords:** Knotted proteins, NMR spectrocopy, protein *trans*-splicing, enzymatic ligation, protein dynamics, protein stability and folding

## Abstract

Knots have attracted scientists in mathematics, physics, biology, and engineering. Long flexible thin strings easily knot and tangle as experienced in our daily life. Similarly, long polymer chains inevitably tend to get trapped into knots. Little is known about their formation or function in proteins despite >1,000 knotted proteins identified in nature. However, these protein knots are not mathematical knots with their backbone polypeptide chains because of their open termini, and the presence of a “knot” depends on the algorithm used to create path closure. Furthermore, it is generally not possible to control the topology of the unfolded states of proteins, therefore making it challenging to characterize functional and physicochemical properties of knotting in any polymer. Covalently linking the amino and carboxyl termini of the deeply trefoil-knotted YibK from *Pseudomonas aeruginosa* allowed us to create the truly backbone knotted protein by enzymatic peptide ligation. Moreover, we produced and investigated backbone cyclized YibK without any knotted structure. Thus, we could directly probe the effect of the backbone knot and the decrease in conformational entropy on protein folding. The backbone cyclization did not perturb the native structure and its cofactor binding affinity, but it substantially increased the thermal stability and reduced the aggregation propensity. The enhanced stability of a backbone knotted YibK could be mainly originated from an increased ruggedness of its free energy landscape and the destabilization of the denatured state by backbone cyclization with little contribution from a knot structure. Despite the heterogeneity in the side-chain compositions, the chemically unfolded cyclized YibK exhibited several macroscopic physico-chemical attributes that agree with theoretical predictions derived from polymer physics.

## Introduction

Knots always fascinate people and have attracted scientists from all disciplines. Long flexible strings can spontaneously knot themselves upon agitation (Raymer and Smith, 2007). Whereas circular supercoiled DNA in nature can be a true mathematical knot, proteins are linear polymers consisting of 20 different amino acids connected by peptide bonds with open amino (N) and carboxyl (C) termini. Proteins fold into defined three-dimensional (3D) conformations and execute various functions at the molecular level. The apparent complexity of threading events involved in tying a protein knot made them inconceivable to many structural biologists at first (Mansfield, 1994). Nevertheless, systematic surveys of the protein database have identified more than 1,000 knotted protein structures with different knot types and structural complexities. However, in this context, “knot” does not imply a topological knot, which cannot be undone except by breaking the protein backbone, a knot in the sense of knotted tie, or a sailor’s reef knot (Jamroz et al., 2015; Lim and Jackson, 2015). It has been challenging to reconcile experimental and theoretical views of how a polypeptide chain attains an intricately knotted topology (Mallam et al., 2008; Mallam and Jackson, 2012; Beccara et al., 2013; Sulkowska et al., 2013; Lim and Jackson, 2015; Ziegler et al., 2016; Jackson et al., 2017). Now, there have been many experimental studies for better understanding of the protein knotting mechanisms. While the majority of experimental studies showed that knotted proteins fold into the knotted conformations with highly populated folding intermediates along their kinetic folding pathways (Mallam and Jackson, 2007; Andersson et al., 2009; Lim and Jackson, 2015; Wang et al., 2015; Lou et al., 2016; Wang et al., 2016; Dabrowski-Tumanski and Sulkowska, 2017; Jackson et al., 2017; He et al., 2019; Jarmolinska et al., 2019; Rivera et al., 2020), there are also some knotted (or slipknotted) proteins that can fold without populating intermediate states (He et al., 2019; Rivera et al., 2020). Different experimental studies have shown that knotting is rate-limiting (Mallam and Jackson, 2012). Computational approaches have also been used to verify the experimental observations, such as the rugged free energy landscapes of several knotted proteins and multiple intermediates populated along their folding pathways. These computational studies might explain the rate-limiting step of protein knotting (Li et al., 2012; Beccara et al., 2013; Sulkowska and et al., 2013; Faísca, 2015) and lead to various protein knotting mechanisms, such as direct threading, slipknotting, and mousetrapping (Noel et al., 2010; Covino et al., 2014). Only very recently, we combined experimental and computational data to obtain a converged view of how the smallest knotted protein, MJ0366, attains a knotted transition state (Passioni et al., 2021). However, all investigations of protein knots have so far been reported for protein knots with open ends because proteins are synthesized as linear polypeptide chains. As such, proteins are not true mathematical knots but are defined by virtual connections of the N- and C-termini by different mathematical schemes (Taylor, 2000; Lai et al., 2012; Millett et al., 2013).

In this study, we asked whether a backbone knotted protein without open ends could be generated by backbone cyclization. Whereas backbone cyclization of proteins has been widely accepted to stabilize proteins (Iwai and Plückthun, 1999; Scott et al., 1999; Clark and Craik, 2010; Montalbán-López et al., 2012; Borra and Camarero, 2013), disulfide bridges, which were originally considered to stabilize proteins by reducing the entropy of the denatured state as backbone cyclization, have more enthalpic contributions to the stability in the folded state (Mitchinson and Wells, 1989; Betz, 1993). Moreover, backbone cyclization of knotted proteins could create the unique possibility to investigate the unfolded state of proteins with a knot unequivocally. A backbone cyclized knotted protein would conform to the mathematical definition for a truly knotted topology whose path closure does not depend on how the ends are joined together in space. Whereas naturally occurring protein knots have open polypeptide-chain ends and could be disentangled into linear polypeptide chains without knots and entanglements under denaturing conditions, backbone cyclized knotted proteins cannot untie anymore without proteolysis even under denaturing conditions.

Here, we presented the unprecedented characterization of a mathematical backbone protein knot without open peptide-chain ends using various structural and biophysical methods including SAXS, X-ray crystallography, and ^15^N nuclear relaxation analysis by NMR spectroscopy.

## Results

### Production of a Knotted Protein With and Without Open Ends

**FIGURE 1:**
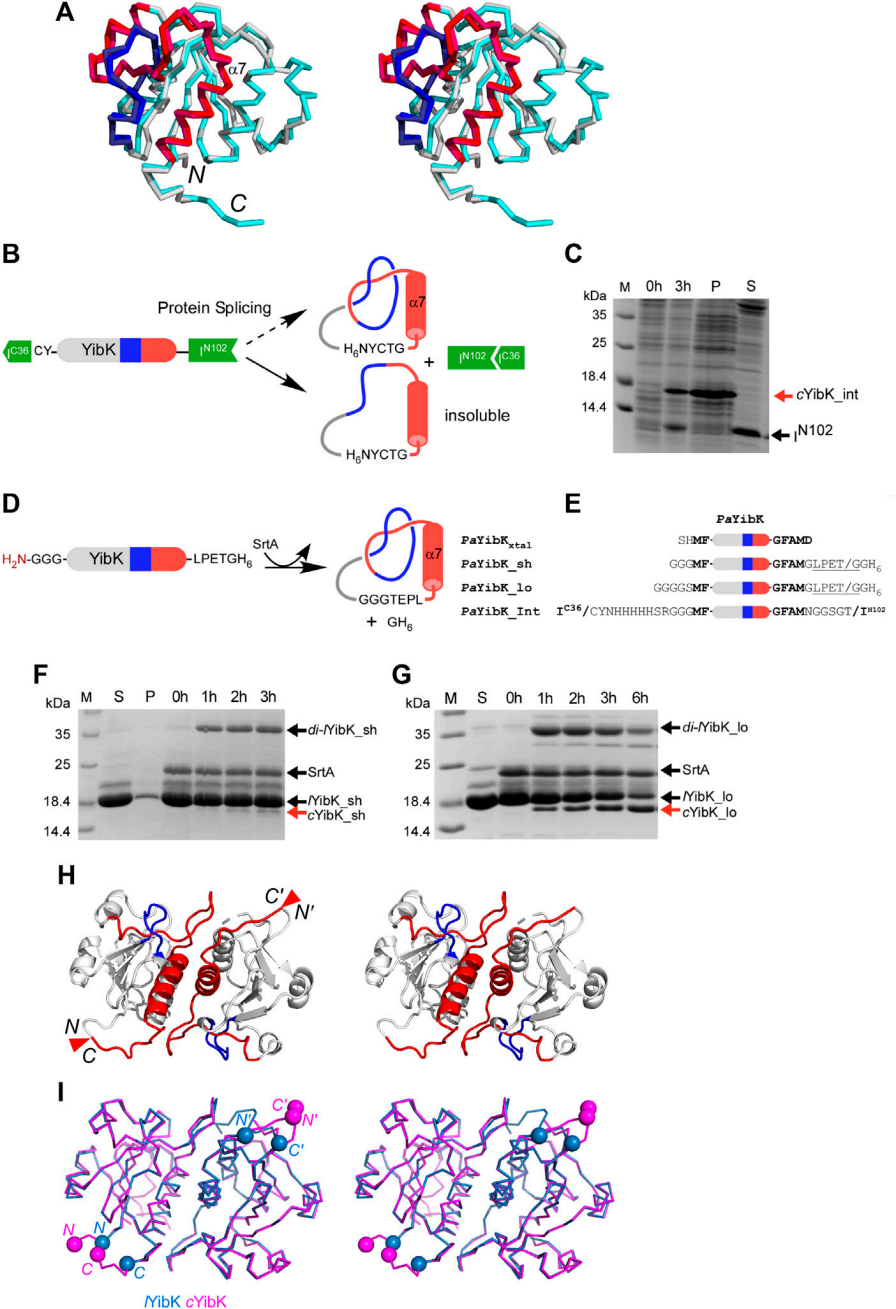
Cyclization and structural analysis of a truly knotted *Pa*YibK. **(A)** A stereo view of the superposition of *Pa*YibK (pdb:6qkv) and *Ha*YibK (pdb:1j85) in ribbon presentation. **(B)** Schematic representation of the experimental strategy used for cyclization of YibK by the split-intein strategy. **(C)** *In vivo* backbone cyclization of YibK by protein *trans*-splicing. Spliced YibK and N-terminal fragment are indicated; M, Molecular weight marker P, insoluble fraction; S, soluble material. **(D)** Backbone cyclization of YibK using sortase A (SrtA). **(E)** Constructs used in backbone cyclization with different lengths of linkers connecting the termini. **(F)** and **(G)** Impact of linker lengths on the *in vitro* sortase ligation efficiency monitored by SDS-PAGE of linear YibK with short **(F)** and long **(G)** poly-glycine linkers annotated as *l*YibK_sh and *l*YibK_lo, respectively; 0–6 h: ligation reactions harvested at specified time points; SrtA, sortase added post-purification; *di-*YibK_sh/lo, undesired dimeric ligation products; *c*YibK_sh/lo, desired cyclized products. The N and C-terminal extensions of YibK for different ligation experiments are shown with their names indicated on the left. **(H)** A stereo-view of the crystal structure of *c*YibK_lo. The structures are shown in cartoon representation with the knotting loop and threading C-terminal helix shown in blue and red, respectively. The positions of the path closures between N- and C-termini are indicated by red triangles. **(I)** Comparison of the crystal structure of linear *Pa*YibK (6qkv; orchid blue) and *c*YibK (6qh8; magenta). The Cα atoms of the S1 and D155 of *Pa*YibK are shown in orchid blue spheres, and the positions of path closures in *c*YibK are indicated by magenta triangles as in **(H)**.

To produce a protein knot without open polypeptide ends, we chose the highly conserved bacterial RNA methyltransferase as a model system, namely YibK from *Pseudomonas aeruginosa* (*Pa*YibK). YibK contains a trefoil (3_1_) knotted backbone topology. *Pa*YibK also shares 65% sequence identity with one of the most-studied knotted proteins, YibK form *Haemophilus influenzae* (*Hi*YibK), but contains only one tryptophan (Supplementary Figure S1). (Tkaczuk et al., 2007) We first determined the crystal structure of the wild-type *Pa*YibK in its linear form (*Pa*YibK) to confirm the same trefoil knot structure and the dimeric assembly as found in *Hi*YibK (Figure 1A; Supplementary Table S1; and Supplementary Figure S1). The root mean square deviation (RMSD) was only 0.6 Å between the crystal structures of *Pa*YibK and *Hi*YibK, indicating a highly conserved higher-order structure among YibK proteins throughout evolution including the knot structure (Figure 1A **)**. The N- and C-termini of *Pa*YibK are separated by ca. 8 Å in the crystal structure, which is sufficiently close for the head-to-tail backbone cyclization without disturbing the backbone knot structure. For backbone cyclization of proteins, various strategies have been established, including intein-mediated protein ligation (or expressed protein ligation), enzymatic protein cyclization using enzymes such as sortaseA (SrtA) and asparagine endopeptidase (AEP), and protein *trans*-splicing (PTS) (Scott et al., 1999; Popp and Ploegh, 2011; Mikula et al., 2017; Iwai et al., 2001). We initially attempted *in vivo* protein cyclization of *Pa*YibK by PTS using the naturally split DnaE intein (*Pa*YibK_Int) (Figure 1B) (Aranko et al., 2013), but the *in vivo* spliced product was insoluble (Figure 1C). PTS-based backbone cyclization relies on the self-association of intein fragments that brings the N- and C-termini together during the protein folding, followed by spontaneous auto-catalytic removal of the intein fragments, thereby achieving backbone cyclization (Scott et al., 1999; Iwai et al., 2001). We speculated that the rapid self-association of the split intein fragments could interfere with the folding and knotting of YibK, thereby resulting in insoluble aggregation. The insoluble spliced product could be backbone cyclized YibK without knotting (*c*YibK_Int) (Figure 1C). Therefore, we used an alternative enzymatic approach with *S. aureus* sortase A (SrtA), which catalyzes a *trans*-peptidase reaction between the LPETG motif and an N-terminal tri-glycine peptide (Figure 1D). (Popp and Ploegh, 2011) When a seven-residue linker (*Pa*YibK_sh) was used to link the N and C-termini through SrtA-mediated ligation (Figure 1E), backbone cyclization was inefficient, and accumulation of a covalent dimer (*di-l*YibK_sh) resulting from intermolecular ligation was more prominent than the monomeric backbone-cyclized form (*c*YibK_sh; Figure 1F). This finding underscores the need to further optimize the linker length for the enzymatic ligation. By introducing a longer nine-residue linker to the backbone cyclization (*Pa*YibK_lo), a much more efficient backbone cyclization was achieved to produce a higher amount of monomeric cyclized YibK (*c*YibK_lo hereafter designated as *c*YibK) than the covalent dimer (*di-Pa*YibK_lo; Figure 1G). We also produced a linear form of YibK (*l*YibK) as the N-terminal SUMO fusion so that *l*YibK and *c*YibK (*Pa*YibK_lo) have the identical protein sequence for the analysis. The backbone cyclization of *c*YibK manifests in greater mobility during SDS-PAGE as compared with linear YibK (*l*YibK); the complete cyclization was also confirmed by mass spectrometry (Supplementary Figure S2).

### Structural Comparison of a Knotted Protein With and Without Open Ends

We determined the crystal structures of *c*YibK to a resolution of 2.20 Å (Figure 1H and Supplementary Table S1). The root mean square deviation (RMSD) between the backbone Cα atoms of the crystal structures of *Pa*YibK and *c*YibK was <0.2 Å, confirming that the backbone cyclization did not perturb the 3D structure of *Pa*YibK in the folded state (Figure 1I). The structural similarity between *l*YibK and *c*YibK and their dimeric states were confirmed by small angle X-ray scattering (SAXS) in solution state (*vide infra*).

### Functional Assessment of *l*YibK and *c*YibK

**FIGURE 2:**
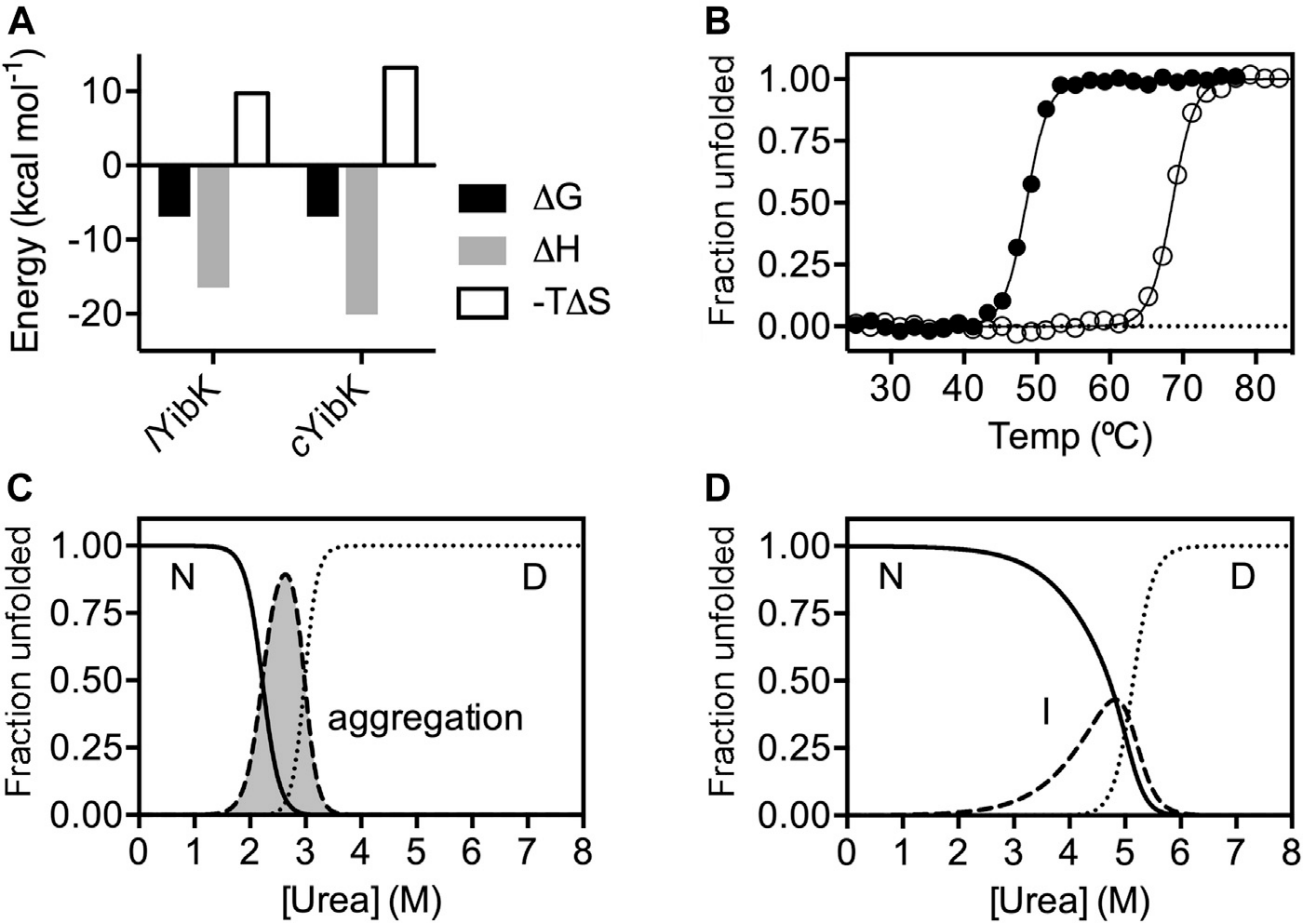
The impacts of cyclization on SAH binding and chemical stability of YibK. **(A)** Thermodynamics parameters associated with SAH binding derived from ITC analyses. **(B)** Normalized fractional unfolded populations of *l*YibK (filled circles) and *c*YibK (open circles) derived from thermal denaturation monitored by far-UV CD spectroscopy. Intrinsic fluorescence-derived normalized fractional populations of native (N), intermediate (I) and denatured (D) states of *l*YibK **(C)** and *c*YibK **(D)** as a function of urea concentration. For *l*YibK, fluorescence signal loss due to aggregation was observed and the corresponding population is shaded in gray and indicated.

Next, we assessed the functional impact of cyclization on the cofactor binding activity, which is essential for the RNA methyltransferase activity of YibK. We used isothermal titration calorimetry (ITC) to determine the dissociation constants (*K*_d_) of *l*YibK and *c*YibK for S-adenosyl-L-homocysteine (SAH), which is the product of the conserved RNA methylation reaction among all SPOUT family members that utilize S-adenosyl-L-methionine (SAM) as the methyl donor (Tkaczuk et al., 2007). The *K*_d_ for SAH was 8.80 ± 0.01 and 8.93 ± 0.03 μM for *l*YibK and *c*YibK, respectively, which corroborates our structural analyses showing no appreciable structural perturbation in *Pa*YibK after the backbone cyclization (Supplementary Table S2; Supplementary Figure S4). In contrast, side-chain disulfide bond-mediated cyclization of *Hi*YibK was reported to decrease SAH binding affinity (*K*_d_ increased from 20 to 71 µM) (Mallam et al., 2010). Furthermore, our experimental *K*_d_ values for SAH binding to *c*YibK*/l*YibK were 2 to 3-fold smaller than the reported values for other tRNA methyltransferases, namely *Hi*YibK (*K*_d_ = 20 μM) (Mallam and Jackson, 2007) and TrmL from *E. coli* (*K*_d_ = 25 μM) (Liu et al., 2013). ITC analysis revealed that SAH binding to *c*YibK was enthalpically more favorable than that of *l*YibK (ΔH = −20.1 vs −16.5 kcal mol^−1^); the difference in enthalpic changes was compensated by the entropic differences (ΔS), resulting in the comparable net free energies of SAH binding (Figure 2A). The greater entropic loss in *c*YibK upon SAH binding may be associated with the dimer formation of *c*YibK.

### Comparison of Folding of *l*YibK and *c*YibK

As *c*YibK and *l*YibK have the identical primary structure and crystal structure in their native states, we assume their associated free energy levels in the folded states are very similar except for the entropic contribution associated with the decreased degrees of freedom of the fraying ends by closing the ends. To investigate how a path closure to form a truly knotted protein may affect folding stability and kinetics, we assessed the thermal stabilities of *l*YibK and *c*YibK by far-UV circular dichroism (CD) spectroscopy and their chemical stabilities by urea-induced chemical denaturation monitored by intrinsic fluorescence. As expected, the apparent melting temperature (*T*_m_) of *l*YibK was increased by 20 °C for *c*YibK (*T*_m_ = 68.7 vs 48.7 °C; Figure 2B). This observation is in line with other proteins with cyclized peptide backbones, suggesting that backbone cyclization of a knotted protein reduced the conformational entropy of the unfolded state. As the thermal unfolding was not fully reversible, particularly in the case of *l*YibK, the *T*_*m*_ values derived from the CD analysis could be underestimated. We additionally analyzed the urea-induced equilibrium unfolding of *l*YibK and *c*YibK (Figures 2C,D). However, we observed the unexpected loss of intrinsic fluorescence of *l*YibK between 2 and 3 M urea, which we attributed to the aggregation of *l*YibK in the analysis **(** Supplementary Figure S4a **)**. In contrast, *c*YibK did not show the similar loss of intrinsic fluorescence during urea-induced denaturation (Supplementary Figure S4b). The experimental data were fit to a three-state unfolding model by the singular value decomposition approach without considering the contributions of dimerization (Wang et al., 2015; Wang et al., 2016). Although the chemical denaturation of *l*YibK was not fully reversible with *l*YibK, the chemical stability of *c*YibK was clearly higher than *l*YibK by >2 M of the transition urea concentration required to unfold *l*YibK and *c*YibK, supporting the increased apparent thermal stability of *c*YibK (Supplementary Table S3; Figures 2C,D).

**FIGURE 3:**
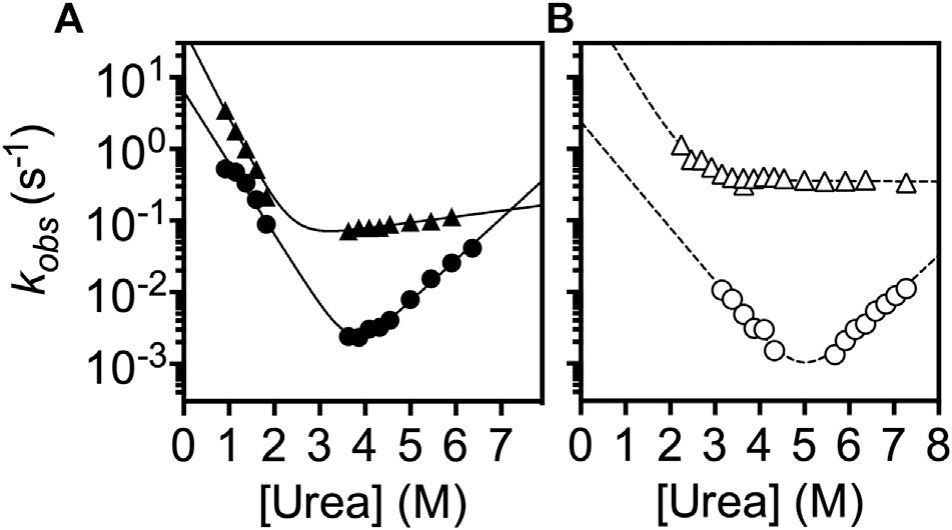
Chevron plot analysis of the folding kinetics of *l*YibK and *c*YibK. The observed folding rates of *l*YibK **(A)** and *c*YibK **(B)** are plotted as a function of urea concentration. Two kinetic phases were observed. Circles and triangles correspond to the slow and fast kinetic phases, respectively. Filled and open symbols are used for *l*YibK and *c*YibK, respectively. The data were fitted to a simple two-state folding model.

Additionally, we analyzed the folding kinetics of *l*YibK and *c*YibK as a function of urea concentration by monitoring the intrinsic fluorescence of the only endogenous tryptophan residue (W150 according to the nomenclature of *l*YibK construct) lining the dimer interface (Figure 3). It is noteworthy that the well-investigated *Hi*YibK contains two tryptophan residues, of which W145 is positioned at the same dimer interface as W150 of *Pa*YibK (Supplementary Figure S4). Similar to the reported multiphasic kinetics of *Hi*YibK, *l*YibK also exhibited two unfolding and refolding phases; the faster phase had a very small *m*-value associated with the unfolding arm (Figure 3). The slower intrinsic unfolding rate of *l*YibK ($k_{u}^{H_{2}O}$) was 9.5 × 10^−6^ sec^−1^, almost 20-fold faster than that of *Hi*YibK ($k_{u}^{H_{2}O}$ = 4.9 × 10^−7^ sec^−1^) (Mallam et al., 2008). The faster unfolding rate of *l*YibK is presumably associated with the aggregation of *l*YibK we observed. The slower intrinsic unfolding phase of *c*YibK ($k_{u}^{H_{2}O}$ = 6.5 × 10^−7^ sec^−1^) was about 7-fold slower than that of *l*YibK (Table 1). The transition urea concentration [D]_50%_, associated with the slower kinetic phases of *l*YibK and *c*YibK agreed well with the second transition points derived from equilibrium unfolding (Figure 2), suggesting that these kinetic phases are associated with the intermediate-to-denatured state transitions. Consequently, the faster kinetic phases of both *l*YibK and *c*YibK would correspond to the intermediate-to-native state transition. Note that the β-Tanford values (β_T_), reporting on the compactness of the transition state with respect to the folded state, were close to 1 for the faster kinetic phase of both *l*YibK and *c*YibK. Thus, the associated transition states (from intermediate to native state; $TS_{I-N}$) could be as compact as the native state (Supplementary Table S3). (Fersht, 1999) In contrast, the β_T_ values of the slower kinetics of *c*YibK were significantly lower (ca. 0.6) for both *l*YibK and *c*YibK, so the corresponding transition state (from denatured to intermediate state; $TS_{D-I}$) is highly disordered (Supplementary Table S3). Collectively, our kinetic analyses suggest that the intermediate formation is the rate-limiting state, which is consistent with the equilibrium-unfolding analysis finding that the intermediate state of *c*YibK was lowly-populated and that the intermediate state of *l*YibK was aggregation-prone.

**TABLE 1:** Kinetic parameters derived from chevron plot analysis of *l*YibK and *c*YibK.

	Kinetic phase	$k_{f}^{H_{2}O}\;$(s^−1^)	*m*_f_ (kcal mol^−1^ M^−1^)	$k_{u}^{H_{2}O}\;$(s^−1^)	*m*_u_ (kcal mol^−1^ M^−1^)	*m*_kin_ ^a^ (kcal mol^-1^ M^-1^)	β_T_ ^b^	[D]_50%,kin_ (M)^c^	Δ*G*_kin_ (kcal mol^−1^)^c^
*l*YibK	Fast	47.6 ± 3.7	−2.87 ± 0.06	0.036 ± 0.002	0.19 ± 0.01	3.06 ± 0.06	0.94 ± 0.05	2.35 ± 0.06	4.25 ± 0.06
	Slow	6.42 ± 1.28	−2.30 ± 0.13	(9.50 ± 4.64) × 10^−6^	1.33 ± 0.09	3.63 ± 0.16	0.63 ± 0.06	3.70 ± 0.26	7.94 ± 0.30
*c*YibK	Fast	124 ± 129	−2.28 ± 0.45	0.360 ± 0.063	∼0^d^	2.28 ± 0.45^e^	1.00^e^	2.56 ± 0.69^e^	3.46 ± 0.62^e^
	Slow	2.41 ± 1.13	−1.71 ± 0.13	(6.55 ± 3.60) × 10^−7^	1.35 ± 0.08	3.06 ± 0.15	0.56 ± 0.05	4.94 ± 0.34	8.95 ± 0.43

a *M*_kin_ = *m*_u_–*m*_f_.

b β_T_ = –*m*_f_/*m*_kin_.

c The transition points ([D]_50%,kin_) and free energies of unfolding (ΔG_kin_) were derived from the kinetic parameters associated with the fast and slow phases.

d The unfolding arm of the slow kinetic phase of *l*YibK showed no apparent denaturant concentration-dependency.

e The results were derived by setting *m*_u_ to zero.

### Comparison Between *l*YibK and *c*YibK Under a Denaturing Condition by NMR

Considering that the chemical compositions (sequences) and 3D structures of *c*YibK and *l*YibK essentially are identical with the exception of a peptide bond introduced, we presumed that the native states of both YibKs have similar free energies. We assume that the observed changes in the folding/unfolding pathway of YibK could be caused by the unfolded state of *c*YibK, which has a significantly reduced conformational space compared with a linear polypeptide due to the circular backbone peptide chain having closed ends and the presence of a knot structure.

**FIGURE 4:**
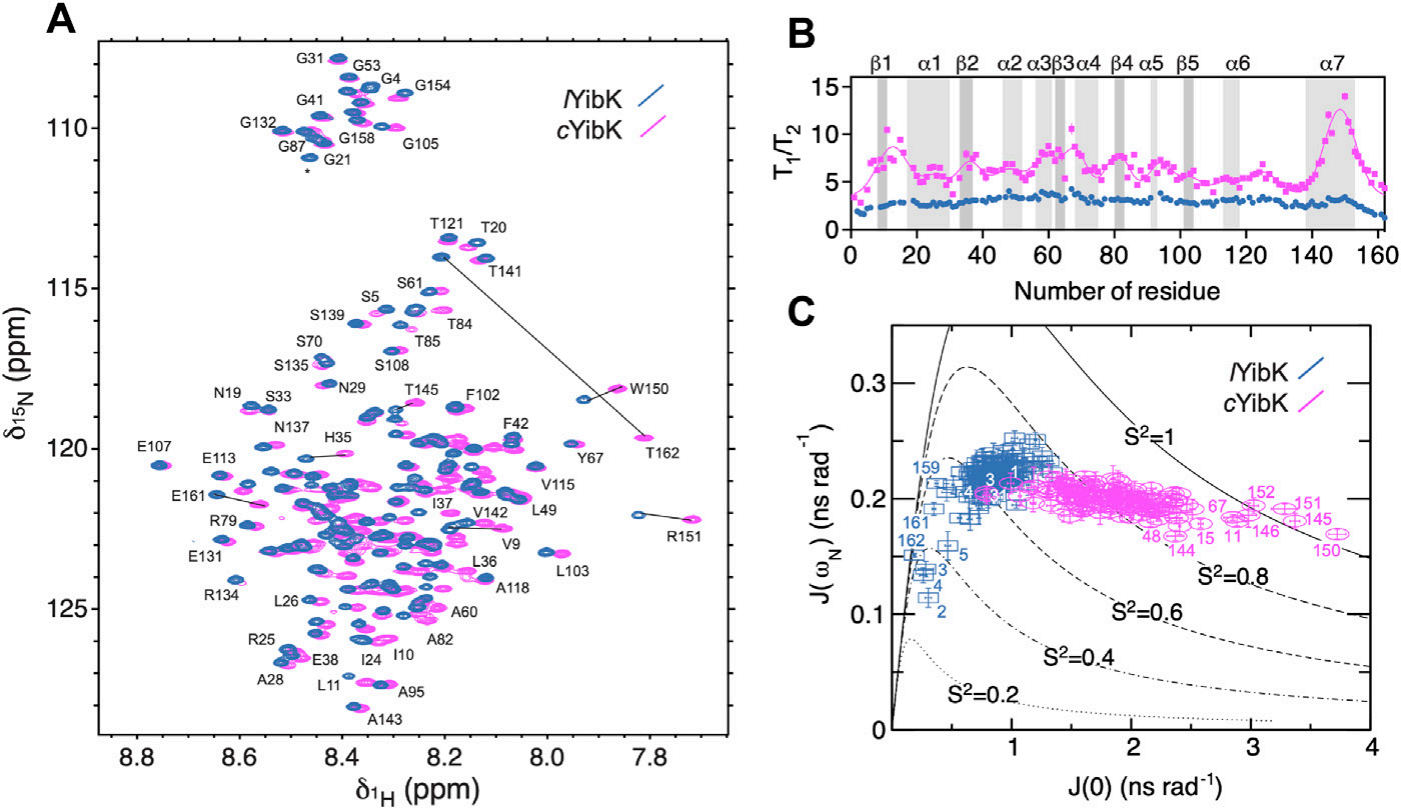
NMR spectroscopy and ^15^N spin relaxation analysis of the chemically denatured *l*YibK and *c*YibK. **(A)** Superimposition of the [^15^N-^1^H] correlation spectra of *l*YibK (orchid blue) and *c*YibK (magenta) in the presence of 7.2 M urea. The spectra were recorded at a ^1^H Larmor frequency of 850 MHz, and 298 K. Residues with large chemical shift differences upon cyclization are indicated by solid lines that connect the pairs of crosspeaks. **(B)** *T*_1_/*T*_2_ ratios of *l*YibK and *c*YibK as a function of residue number. *T*_1_/*T*_2_ ratios of *c*YibK were fitted to a sum of multiple Gaussian distributions. Regions that correspond to the β-sheets and α-helices in the native structure are highlighted by dark and light gray, respectively, and are indicated above the panel. **(C)** Spectral density mapping of the ^15^N relaxation data expressed as *J* (ω_N_) as a function of *J* (0). Residues that deviate from the cluster distributions are indicated with their residue numbers. Theoretical curves with the assumption of isotropic motions were calculated for different-order parameters, S (Liu et al., 1980), as indicated. All data points are colored using the same scheme as in (**A**).

As NMR spectroscopy can investigate proteins under various solution conditions including denaturating ones, we used NMR to characterize the unfolded states into both *l*YibK and *c*YibK in 7.2 M urea to gain structural insights into the denatured states of *l*YibK and *c*YibK (Figure 4). The two-dimensional [^15^N-^1^H] correlation spectra for both *l*YibK and *c*YibK in 7.2 M urea showed poor chemical shift dispersions along the ^1^H dimension, characteristic of unfolded and disordered polypeptides (Figure 4A). Furthermore, a large number of crosspeaks exhibited major chemical shift differences between the two [^15^N-^1^H] SOFAST-HMQC spectra (Figure 4A). We could obtain near-complete site-specific NMR assignments of the observed [^15^N-^1^H] correlations of *l*YibK and *c*YibK in 7.2 M using a described protocol (Hsu et al., 2009; Hsieh et al., 2014). As expected, the backbone amide of T162 at the C-terminus of *l*YibK showed the largest chemical shift difference from that of *c*YibK owing to the introduction of an additional peptide bond as a result of backbone cyclization. Furthermore, several residues near the N- and C-termini also exhibited significant chemical shift perturbations (Figure 4A; Supplementary Figure S6).

NMR chemical shift analysis suggested very limited structural differences between urea-denatured *l*YibK and *c*YibK (Supplementary Figure S6). Therefore, we performed ^15^N relaxation analysis to compare their backbone dynamics. Under the denaturing condition, the mean ^15^N transverse relaxation time (*T*_2_) for *c*YibK was much shorter than that of *l*YibK in 7.2 M (122 vs. 238 ms; Supplementary Figure S7). We also observed that *l*YibK exhibited longer and more uniform *T*_2_ values across the primary sequence with the exception of the fraying ends because of the unrestricted chain dynamics at the open ends Supplementary Figure S7C). In contrast, *c*YibK exhibited clusters of fast-relaxing residues across the primary sequence (Supplementary Figure S7C), reminiscent of the previously observed long-range non-native interactions in urea-denatured lysozyme (Klein-Seetharaman et al., 2002). For urea-denatured lysozyme, the non-native interactions reflected the clustering of bulky hydrophobic tryptophan residues. However, for *c*YibK, not all rapidly relaxing residues contain aromatic side-chains. The *T*_2_ relaxation clusters may stem from the internal friction imposed by backbone cyclization that restricted the fraying motions of the N- and C-termini. Differences in dynamics or populations were particularly manifested in the dispersed *T*_1_/*T*_2_ values for *c*YibK, whereas a flat profile of a *T*_1_/*T*_2_ values was observed for *l*YibK. The *T*_1_/*T*_2_ values indicated that the differences of backbone dynamics are in the timescale of micro-to milliseconds. In contrast, the heteronuclear ^15^N{^1^H}-NOE of *l*YibK and *c*YibK, which is sensitive to a faster pico-to-nanosecond timescale, did not show apparent differences except for the termini of *l*YibK (Supplementary Figure S7c). Thus, the backbone cyclization did not affect the pico-to-nanosecond motions of the individual backbone peptide bonds except for the termini with the conformation restriction imposed by backbone cyclization (Supplementary Figure S7).

We also applied the reduced spectral density mapping approach for studying the dynamics of both denatured *l*YibK and *c*YibK (Figure 4C). (Farrow et al., 1995; Peng and Wagner, 1995; Shih et al., 2015) The results identified two distinct clusters of spectral density distributions for urea-denatured *l*YibK and *c*YibK, the former located around the theoretical curve for an order parameter *S* (Liu et al., 1980) of <0.7 and the latter exhibiting a cluster around the *S* (Liu et al., 1980) value of 0.8 (Figure 4C). Furthermore, several residues located at the C-terminal helix (α7) and some others were located outside the cluster distribution. Collectively, the NMR relaxation dynamics analysis suggested the presence of abundant conformational exchanges. It also implied a broad range of backbone dynamics caused by the backbone cyclization of YibK, restricting the backbone motions and increasing the ruggedness of the free energy landscape of the denatured state of *c*YibK.

### *c*YibK_Int Under the Denaturing Condition by NMR

The split-intein approach for cyclization of YibK resulted in the insoluble spliced product that could presumably be the cyclized YibK without knotting (*c*YibK_Int) (Figure 1C). Even though *c*YibK_Int has an additional hexahistidine-tag and slightly different amino-acid sequence connecting the N- and C-termini (Figure 1E), we decided to purify and investigate *c*YibK_Int under a denaturing condition by NMR spectroscopy (Supplementary Figure S8). ^15^N{^1^H}-NOE data for the N- and C-termini of *c*YibK_Int are similar to those of *c*YibK than *l*YibK bearing flexible termini due to the linear polypeptide chain. This observation confirms the backbone cyclization of *c*YibK_Int. The average *T*_2_ relaxation time for *c*YibK_Int was shorter than that of *l*YibK (157 vs. 238 ms) (Supplementary Figure S8c). On the other hand, a flat profile of *T*_1_/*T*_2_ values for *c*YibK_Int is closer to *l*YibK, indicating the absence of conformational exchanges observed with *c*YibK, presumably because of the absence of a knot structure under the denaturing condition (Supplementary Figure S8C).

### Small-Angle X-Ray Scattering Analysis of the Unfolded States of *l*YibK and *c*YibK

**FIGURE 5:**
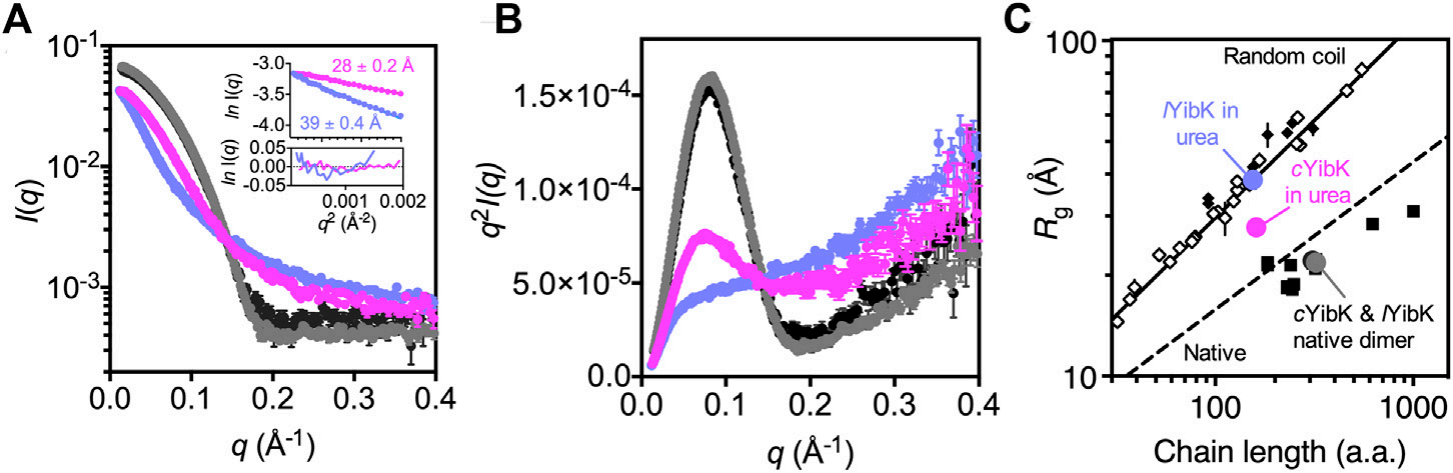
Cyclization significantly reduces the global dimension of the denatured state of *c*YibK. **(A)** SAXS profiles of *l*YibK in 0 M urea (gray), *c*YibK in 0 M urea (black), *l*YibK in 7 M urea (orchid blue), and *c*YibK in 7 M urea (magenta). Inset: Guinier plots of *l*YibK and *c*YibK in 7 M urea with the corresponding *R*_g_ values indicated and fitting residues shown below. **(B)** Kratky plots of *l*YibK and *c*YibK in their native and 7 M urea-denatured states, as shown in (a). Urea-denatured *c*YibK exhibits a distinct bell-shape profile, indicative of compact residual structures that are absent in urea-denatured *l*YibK. **(C)** *R*_g_ values of *l*YibK and *c*YibK in their native and 7 M urea-denatured states as a function of chain length. The solid and dashed lines indicate the experimentally derived scaling relationship for random-coil and native proteins, respectively. Solid squares and diamonds correspond to reported *R*_g_ values of knotted proteins of different topologies and chain lengths. Open diamonds correspond to reported random-coil *R*_g_ values of chemically denatured unknotted proteins (Shih et al., 2015).

We previously used SAXS to demonstrate that chemically denatured knotted proteins with open ends exhibited random coil-like behaviors: their radii of gyration (*R*_g_) scale with their chain lengths to the power of 3/5 (Shih et al., 2015). This observation suggests that backbone knotting with open ends does not necessarily lead to significant compaction of the overall chain dimension under highly denaturing conditions (in good solvent). However, these knotted proteins examined by SAXS are not mathematically knotted because of their free termini. To examine how backbone cyclization may affect the polymer properties of a mathematically knotted protein, we compared the SAXS data of *l*YibK and *c*YibK by using online size-exclusion chromatography-coupled SAXS (SEC-SAXS) apparatus as described (Lee and Hsu, 2018). Under native conditions, *l*YibK and *c*YibK exhibited the same SAXS profiles, with comparable *R*_g_ values — 22.17 ± 0.04 and 21.66 ± 0.06 Å—that were in general agreement with the theoretical value based on the crystal structure (19.4 Å; Figure 5). The corresponding Kratky plots showed comparable compactness (similar bell-shape profiles; *cf*. black and gray curves for *c*YibK and *l*YibK in Figure 5B, respectively). In contrast, 7.2 M urea-denatured *c*YibK showed a significantly smaller *R*_g_ value than *l*YibK under the same condition (27.71 ± 0.15 vs 39.26 ± 0.38 Å; Figures 5A,C). Urea-denatured *l*YibK exhibited a monotonously increasing Kratky profile typical of a random coil polypeptide, whereas urea-denatured *c*YibK exhibited a distinct bell-shape profile, albeit much smaller than that of folded *c*YibK, which indicates the presence of compact residual structure (blue curve in Figure 5B). According to the empirical scaling relationship established from our previous study, the expected *R*_g_ value for a chemically denatured *l*YibK is 38.0 Å, assuming a random coil-like behavior (Figure 5C). (Shih et al., 2015) A reduction of *R*_g_ by >10 Å in chemically denatured *c*YibK equals a decrease of the global dimension by 1/4 and a 3/5 decrease of the excluded volume (assuming that the exclusion volume of the unfolded polypeptide chain is spherical). The substantial conformational compaction further suggests that the conformational entropy of the unfolded state is reduced as a result of the backbone cyclization, which is in line with the stability improvement of *c*YibK.

## Discussion

Introduction of a peptide bond between N- and C-termini into a knotted protein unambiguously converts it to a truly mathematical knot without the need to evoke convoluted knot-detecting algorithms, which in some cases have different interpretations of “knots” in proteins. It also eliminates any confusing experimental effects due to fraying of the N- and C-termini in proteins that are “knotted” by the introduction of disulfide bridges, which are largely different due to higher rotational degrees in the side-chains (Betz, 1993; Mallam et al., 2010). Whereas disulfide bonds have a mixture of enthalpic and entropic effects on the protein stability, backbone cyclization connecting the N- and C-termini is generally accepted to stabilize protein by destabilizing the unfolded state (Betz, 1993; Iwai and Plückthun, 1999; Scott et al., 1999; Clark and Craik, 2010; Montalbán-López et al., 2012; Borra and Camarero, 2013).

Here, we produced a trefoil-knotted protein without open ends, a truly mathematical backbone knot in a protein, by post-translational modification by enzymatic ligation (Figure 1). Such a backbone modification is irreversible as opposed to disulfide crosslinking between a pair of engineered cysteines at the N- and C-termini. Furthermore, the path closure by a backbone peptide bond essentially removes the origin of the protein sequence, rendering obsolete the conventional definition of a protein folding topology by the hierarchical arrangements of secondary structure elements. As compared with circular permutation, which was recently used to untie the trefoil-knotted *Hi*YibK (Chuang et al., 2019), and *E. coli* YbeA (Ko et al., 2019), SrtA-mediated backbone cyclization allowed us to examine the contribution of a true backbone knot from a completely different perspective with clarity. The apparent melting temperature was increased by 20  °C for the knotted *c*YibK compared with *l*YibK with open ends (Figure 2). The path closure by a peptide bond after folding also has seemingly remodeled the protein folding/unfolding pathway of the original *l*YibK and alleviated the aggregation propensity of the folding intermediate observed for *l*YibK, whereas maintaining the native structure and ligand binding affinity (Figure 2; Supplementary Table S2). Indeed, the backbone cyclization significantly increased the folding rate of the intermediate-to-native state transition, with the corresponding transition state being highly compact and native-like, as evidenced by the β_T_ value being close to 1 (Figure 3; Supplementary Table S3). Furthermore, the unfolding rate of intermediate-to-denatured state transition of *c*YibK, $k_{u}^{H_{2}O}$ (derived from the slower kinetic phase; Figure 3) was >10 times slower than that of *l*YibK, so the denatured state of *c*YibK may have a status with higher Gibbs energy than that of *l*YibK, which could be supported by *R*_g_ in 7 M urea estimated by the SAXS data. Note that we have not unambiguously established whether *l*YibK is unknotted or not under urea-denatured state as has been demonstrated earlier (Mallam et al., 2008; Capraro and Jennings, 2016). It is, therefore, possible that the denatured *l*YibK may exist in a mixture of knotted and unknotted structures.

To this end, ^15^N spin relaxation analysis of *l*YibK showed no appreciable conformational exchange contributions to the three different timescales probed by *J* (0), *J* (ω_H_), and *J* (ω_N_), suggesting the absence of interconversion between knotted and unknotted states of *l*YibK (Supplementary Figure S7). *c*YibK_Int, which has closed peptide ends and possibly no knot structure, showed a similar profile of *T*_1_/*T*_2_ to that of *l*YibK in the ^15^N spin relaxation analysis. In contrast, the enhanced *T*_2_ relaxation observed in *c*YibK, which has significant contributions to the *J* (0) term, likely reflects the increased internal friction of the cyclized polypeptide chain in the denatured state, thereby leading to destabilization of the unfolded state (Figure 4). As observed for many backbone cyclized proteins, we think that the increase in folding stability of *c*YibK could be attributed mainly to the reduced conformational entropy in the denatured state of *c*YibK.

In line with the stability enhancement, the chemically denatured state of *c*YibK was significantly more compact than the random coil-like *l*YibK in 7 M urea (Figure 5). The effect of cyclization on the polymer dimension is well understood in the literature. Kramers’ polymer model predicts that the *R*_g_ values of linear and cyclized polymers follow a simple relationship.

$$\;\frac{{\langle R_{g}^{c}\rangle }^{2}}{{\langle R_{g}^{l}\rangle }^{2}}=\frac{1}{2}$$

where $\langle R_{g}^{c}\rangle$ and $\langle R_{g}^{l}\rangle$ are the mean *R*_g_ values of the cyclized and linear forms of the same polymer, respectively, (Kramers, 1946). Our SAXS analysis of *c*YibK and *l*YibK yielded a ratio of 0.50 ± 0.01, which is in good agreement with Kramers’ polymer theory despite the highly heterogeneous amino acid side-chain compositions (Kramers, 1946). The SAXS data implied that a knot formation does not provide any further compactness under a denaturing condition by having a true knot structure after backbone cyclization. In other words, there might be no or little entropic penalty for knotting under highly denaturing conditions due to the high flexibility, suggesting that the stability enhancement of *c*YibK could be attributed mainly to backbone cyclization without additional contribution from the knot structure. The SAXS analysis suggested that the cyclized and knotted YibK under a highly denaturing condition appears to comply with the polymer physics developed for non-self-interacting Gaussian chains, behaving like a long thin string. If this is true for unfolded proteins without denaturants (i.e., intrinsically disordered proteins), some proteins without defined secondary structures could possibly entangle into open knots at a certain probability, as observed for simpler homopolymers (Higgins et al., 1979; Arrighi et al., 2004). An increasing number of intrinsically disordered proteins without fixed conformations have been identified and implicated in many diseases. Our results suggest that protein backbone knots could also be transiently formed without any entropic penalty when polypeptide chains are very flexible, as in the denatured states. Physico-chemical characterizations of even simpler polymers with a well-defined mathematical knot have not been investigated because isolating defined simple polymers with a specific mathematical knot is very challenging. Unlike other simpler polymers, the self-entanglement of proteins into defined knots could be exploited to isolate well-defined mathematical knots for further physicochemical characterizations. The post-translational enzymatic backbone cyclization, as well as the split-intein approach we demonstrated here, could pave the way to investigate other proteins with various knot topologies, which may include transiently formed protein knots, for example, with intrinsically disordered proteins.

## Materials and Methods

### Constructions and Production of Recombinant YibK Variants

For backbone cyclization, *P. aeruginosa* YibK with a sortase recognition sequence LPETG followed by the C-terminal hexa-histidine (H_6_) was cloned in pRSF vector as N-terminal SUMO fusion by PCR, resulting in pITRSF1A (*Pa*YibK_sh) and pITRSF3D (*Pa*YibK_lo). *In vivo* cyclization vector for *Pa*YibK was cloned into a pBAD vector containing the genes of split *Npu*DnaE-C intein fragment, H_6_-tag, and *Npu*DnaE-N intein fragment by using *Xba*I/*Kpn*I sites, resulting in pJMBAD36(*Pa*YibK_Int) (Iwai et al., 2006). For biophysical characterization of linear *Pa*YibK_lo, plasmid pBHRSF260 was constructed with N-terminally His-tagged SUMO fusion to have the identical sequence as *Pa*YibK_lo construct (Guerrero et al., 2015). Each plasmid was transformed into *E. coli* strain ER2566 cells (New England Biolabs, Ipswich, United States). The cells were cultured in Luria-Bertani medium supplemented with kanamycin at 25 μg⋅ml^−1^ until OD_600nm_ reached 0.6 at 37 °C. The recombinant protein overexpression was induced for 4 h with a final concentration of 1 mM IPTG. The cells were harvested by centrifugation and resuspended in binding buffer (50 mM sodium phosphate buffer, pH 8.0, and 300 mM NaCl). The resuspended cells were lyzed at 15,000 psi for 10 min by using Emulsiflex C3, and the supernatant was separated from cell debris by 1 h centrifugation at 38,465 *g*. The supernatant was loaded onto a pre-packed HisTrap HP column (GE Healthcare Life Sciences, United States). The His-tagged fusion proteins were eluted by a linear gradient of 50–250 mM imidazole and dialyized against 2 L of 50 mM Tris-HCl buffer, pH 7.5, 0.5 mM EDTA, and 0.5 mM DTT overnight at 8 °C (Guerrero et al., 2015). The fusion proteins were digested by Ulp1 protease as described previsouly (Guerrero et al., 2015). The digested fusion proteins were loaded again on pre-equibriated HisTrap HP column and washed to remove the SUMO-tag. The C-terminally His-tagged YibK were eluted by a linear gradient of 50–250 mM imidazole and dialyzed against 50 mM Tris-HCl buffer, pH 7.5 overnight, followed by concentration with a centrifugal device. *c*YibK by the split intein fusion (*c*YibK_Int) was produced from the plasmid pJMBAD36. *E. coli* strain ER2566 cells bearing pJMBAD36 were grown in 2 L of M9 medium supplemented with ampicilin at 100 μg⋅mL^−1^ at 37 °C and induced for 4 h with a final concentration of 0.02% (w/v) arabinose. The cells were harvested and lyzed at 15,000 psi for 10 min using Emulsiflex C3. The insoluble pellet was collected after discarding the supernatant by 1 h centrifugation at 38,465 *g*. The pellet was resolubilized in 25 ml of 8M urea with shaking at 350 rpm overnight. The dissolved solution was cleared by 1 h centrifugation at 38,465 *g*. The supernatant was loaded onto the HisTrap HP column, which was pre-equilibrated with a binding buffer (100 mM sodium phosphate buffer, pH 8.0, 10 mM Tris, and 8 M urea). The His-tagged cyclized YibK (*c*YibK_Int) was eluted by 100 mM sodium phosphate buffer, pH 5.0, 10 mM Tris, and 8 M urea. The precursor protein in the elution fractions was removed by size-exclusion chromatography with a Superdex 75 16/60 column (GE Healthcare, United States) in 20 mM sodium phosphate buffer, pH 5.0, 8M urea. The fractions containing *c*YibK_Int were pooled and concentrated for NMR analysis.

### SrtA-Mediated Backbone Cyclization

For the backbone cyclization, sortase (SrtA, from *Staphylococcus aureus*) was added to purified YibK in 1–5 molar ratio and dialyzed against 50 mM Tris-HCl buffer (pH 7.5), 10 mM CaCl_2_ and 2 mM DTT, for 20 h at room temperature. Finally, the unreacted YibK that contained the His_6_-tag at the C-terminus was separated from *c*YibK by incubation with Ni-NTA resin in an open column. The *c*YibK collected from the flow-through fractions was further polished by size-exclusion chromatography with a Superdex 75 16/60 column (GE Healthcare, United States) in buffer A (50 mM Tris-HCl (pH 7.4), 0.5 mM EDTA and 5 mM DTT).

### Protein Crystallography

For the crystal structure of *Pa*YibK, the plasmid (pJMRSF13) encoding YibK gene with N-terminal His-tag and the SUMO fusion was produced and purified as described previously (Guerrero et al., 2015). Crystallization was performed with 9.2 mg/ml solution of *Pa*YibK and 11 mg/ml solution of *c*YibK. Drops of 200 nl (100 nl protein solution and 100 nl well solution) were placed in 96-well MRC (Molecular Dimensions) crystallization plates using a Mosquito LCP (TTPLabtech, United Kingdom). Initial hits were obtained from the traditional sparse matrix screens with the local modifications (Cudney et al., 1994). The initial hits were further optimized by grid screening. The final growth conditions for diffracting crystals were 0.15 M ammonium sulfate, 0.9 M lithium sulfate, 0.1 M sodium citrate buffer (pH 5.6) for *Pa*YibK, and 0.3 M ammonium sulfate, 0.1 M MES buffer (pH 6.0), 25% polyethylene glycol monomethyl ether (PEG MME) 5,000 for *c*YibK. 20% glycerol was added on top of the drop of *Pa*YibK for cryoprotection prior to flash-freezing crystals in liquid nitrogen. For *c*YibK, the 25% PEG MME 5000 present in the crystallization drop served as a sufficient cryoprotectant. Diffraction data for the crystals of *Pa*YibK and *c*YibK were collected at the beamline ESRF ID14–4, Grenoble, France and I03 at the Diamond Light Source, Oxfordshire, UK, respectively. The diffraction data were then indexed, integrated, and scaled to 2.0 and 2.2 Å resolution for *Pa*YibK and *c*YibK, respectively, in XDS (Kabsch, 2010). The final crystal parameters and data processing statistics are in Supplementary Table S1. The structures of *Pa*YibK and *c*YibK were solved by molecular replacement with MolRep from the CCP4 package (Winn et al., 2011). The structure of *Hi*YibK (PDB ID: 1mxi) was used as a search model for molecular replacement. The model was then built using Coot, followed by rounds of refinement with Refmac5 from the CCP4 package and Phenix (Adams et al., 2010). The final refinement was performed with Phenix, and the quality of the final model was validated by using MolProbity (Supplementary Table S1). (Chen et al., 2010) The final refined model of *Pa*YibK was used as a starting model for the molecular replacement to solve the structure of *c*YibK. The structure was solved, refined and validated as mentioned above (Supplementary Table S1). The final coordinates were deposited in the Protein Data Bank (PDB) with the accession codes 6qkv and 6qh8 for *Pa*YibK and *c*YibK, respectively.

### Chemical Denaturation Monitored by Intrinsic Fluorescence Spectroscopy

Urea-induced equilibrium unfolding of *l*YibK and *c*YibK was monitored by intrinsic fluorescence as described (Wang et al., 2015; Wang et al., 2016). Briefly, 41 aliquots of protein solution (at a final concentration of 2 μM buffered in buffer A) were prepared in a series of urea concentrations (0–7 M) with a linear increment step of 2.5% generated by a two-channel liquid syringe dispenser (Hamilton, United States). The samples were incubated at 25 °C overnight before fluorescence measurements with a fluorimeter (JASCO FP8500, Japan). The samples were excited at 280 nm and emission spectra between 300 and 450 nm were collected. The results underwent singular value decomposition analysis with MatLab (MATLAB and Statistics Toolbox release 2012b; The MathWorks, United States) to determine the number of states associated with the unfolding processes, followed by fitting to a three-state folding equilibrium model with Prism (GraphPad, United States) as described (Wang et al., 2014; Wang et al., 2015; Wang et al., 2016; Lee et al., 2017).

### Thermal Denaturation Monitored by Far-UV CD Spectroscopy

The protein solutions were diluted to 10–15 μM in buffer A with a total volume of 0.3 ml, and transferred into a 1 mm path-length quartz cuvette (Hellma, Germany) for far-UV CD measurements. The CD signals between 195 and 260 nm were collected as a function of temperature between 25 and 80 °C with an interval of 2°C by using a CD spectrometer (J-815, JASCO, Japan). The spectra bandwidth was set to 1 nm with a data interval of 0.5 nm, and an averaging time of 1 s. The melting temperatures (*T*_m_) of *l*YibK and *c*YibK were derived by global-fitting the CD spectra as a function of temperature to a two-state model as described (Wang et al., 2014; Lee et al., 2017).

### Isothermal Titration Calorimetry

ITC analysis of SAH binding to *l*YibK and *c*YibK was monitored by using MicroCal VP-ITC (Malvern, United Kingdom) as described (Zhao et al., 2015). Stock solutions of YibK variants were dialyzed overnight against buffer B (50 mM Tris-HCl (pH 7.4), 0.5 mM EDTA, and 0.1 mM TCEP) to remove DTT before ITC measurements. The dialysis buffer was used to prepare the stock solution of the titrant, SAH (Sigma-Aldrich, United States) at a concentration of 0.5 mM. An amount of 20 μM *l*YibK or 13 μM *c*YibK was used in the sample cells for ITC measurements. The resulting isotherms were processed by using NITPIC followed by data fitting with SEDPHAT (Zhao et al., 2015).

### Folding Kinetics Monitored by Intrinsic Fluorescence Spectroscopy

Chevron plot analyses of the folding kinetics of *l*YibK and *c*YibK involved using a combination of stopped-flow and manual mixing measurements as described (Wang et al., 2015; Wang et al., 2016). Briefly, 10 μM native or 7 M urea-denatured protein stock solution was mixed with 10-fold excess denaturing or refolding buffer (buffer A with different concentrations of urea) and the kinetic traces of total fluorescence emission excited at 280 nm and cutoff by a 320 nm cutoff filter were fit to a linear combination of 2–4 exponential functions depending on the experimental conditions. For the slowest refolding rate of *c*YibK, manual mixing of 7 M urea-denatured *c*YibK with refolding buffer at a 1:10 mixing ratio was performed before fluorescence measurement (excitation 280 nm and emission 325 nm with a bandwidth of 5 nm) with a fluorimeter (JASCO FP8500, Japan).

### Small-Angle X-Ray Scattering

SEC-SAXS experiments were performed on beamline BL23A at the National Synchrotron Radiation Research Center (NSRRC, Hsinchu, Taiwan) with the capacity to separate aggregated particles on a silica-based size-exclusion column (Bio SEC-3, Agilent, United States). SAXS signals were detected by using a Pilatus detector (1M-F) and processed by an in-house developed program to obtain the SAXS profiles (Kohn et al., 2004; Jeng et al., 2010; Lee and Hsu, 2018). The SAXS data were collected for momentum transfer *q* ranging from 0.005 to 0.434 Å^−1^, with X-ray wavelength 1.03 Å and 13 keV. The beam geometry was set to 0.5 × 0.5 mm^2^. During the HPLC separation before SAXS measurements, the mobile phase consisted of buffer A (with and without 7 M urea) with the addition of 2% glycerol to prevent radiation damage. The protein solutions were concentrated to 10 mg/ml with the same mobile phase buffer immediately before SAXS measurements.

### Nuclear Magnetic Resonance Spectroscopy

Uniformly ^15^N-labeled and 20% ^13^C-labeled *c*YibK and *l*YibK were prepared by using M9 medium containing 1 g/L [^15^N] ammonium chloride as a nitrogen source and a mixture of 0.6 g/L [^13^C] D-glucose and 2.4 g/L [U-^12^C] D-glucose as a carbon source, as described (Iwai and Fiaux, 2007; Heikkinen et al., 2021). The NMR samples were fully denatured by 7.2 M urea in buffer A containing 10% D_2_O (v/v) at a protein concentration ca. 0.3 mM. A suite of triple resonance experiments in addition to the [^15^N-^1^H] band-selective optimized flip-angle short transient heteronuclear multi-quantum correlation (SOFAST-HMQC) were recorded at 298 K on 850 MHz NMR spectrometer equipped with a cryogenic triple resonance probe (Bruker, Germany) for backbone resonance assignments following the strategy described previously (Hsu et al., 2009; Hsieh et al., 2014). Near-complete backbone resonance assignments (H^N^, N, C’, Cα, Cβ, and Hα) were achieved for both *c*YibK and *l*YibK. The assignments were deposited in the Biological Magnetic Resonance Bank (BMRB) under accession numbers 27685 and 27686 for *c*YibK and *l*YibK, respectively. ^15^N spin relaxation NMR measurements for longitudinal (R_1_) and transverse (R_2_) relaxation rates and the heteronuclear Overhauser effect (hetNOE) were as described (Hsu et al., 2009). Eight longitudinal relaxation delays (20, 60, 120, 200, 300, 500, 800, and 1,000 ms) and nine transverse relaxation delays (16, 32, 64, 96, 128, 160, 192, 224, and 256 ms) were used for both *c*YibK and *l*YibK, and the data were collected as pseudo-3D spectra with the relaxation delays incremented in an interleaved manner to minimize heating effects. The R_1_ and R_2_ rates were extracted by fitting the peak intensities of the individual residues in the ^15^N-^1^H correlation spectra to a single exponential decay function by using the relaxation analysis module in Sparky (T. D. Goddard and D. G. Kneller, SPARKY 3, University of California, San Francisco, United States).

## Data Availability

The datasets presented in this study can be found in online repositories. The names of the repository/repositories and accession number(s) can be found below: http://www.wwpdb.org/, 6qkv, 6qh8; https://bmrb.io/, 27685, 27686.
