# Supplementary material for: Tying up the Loose Ends: A Mathematically Knotted Protein
Source: Front Chem. 2021 May 24;9:663241. doi: 10.3389/fchem.2021.663241 (PMC8182377; doi:10.3389/fchem.2021.663241)
Supplement: Supplementary file 1 [file DataSheet1.PDF]

Tying up the loose ends: a mathematically knotted protein

Shang-Te Danny Hsu[1,2*], Yun-Tzai Cloud [Lee1,2, ¶], Kornelia M. Mikula[3, ¶], Sofia M. Backlund[3], Igor Tascón[3, #], Adrian Goldman[5] and Hideo Iwaï[3,*]

1. Institute of Biological Chemistry, Academia Sinica, Taipei 11529, Taiwan
2. Institute of Biochemical Sciences, National Taiwan University, 106, Taiwan
3. Institute of Biotechnology, University of Helsinki, P.O. Box 65, Helsinki, FI-00014, Finland
4. Division of Biochemistry, Department of Biosciences, University of Helsinki, Helsinki, FIN-00014, Finland.
5. Astbury Centre for Structural Molecular Biology, School of Biomedical Sciences, University of Leeds, Leeds LS2 9JT, England.

*Correspondence to sthsu@gate.sinica.edu.tw and hideo.iwai@helsinki.fi

Supporting information

Supplementary Tables 3
Supplementary Figures 8
Supplementary references 7

Table S1. Details of crystallographic data collection and refinement statistics

	***Pa*YibK**	***c*YibK**
**Data collection**	ESRF ID14-4	Diamond i03
Wavelength	0.9795 Å	0.9763Å
Space group	$C222_1$	$P2_12_12_1$
Molecules/a.u.	2	4
Unit cell		
a, *b*, *c* (Å);	85.7, 167.1, 48.9	48.9, 85.7, 167.1
α=β=γ (°)	90	90
Resolution (Å)*	2.01 (2.13-2.01)	2.20 (2.33-2.20)
R_{meas} (%)	7.1 (66.5)	13.4 (126.8)
No. of reflections measured/unique	139540/23919 (18839/3813)	479743/36646 (72946/5693)
$\langle I/\sigma I \rangle$	18.52 (2.84)	15.40 (2.24)
Completeness (%)	99.5 (99.2)	99.5 (97.3)
Redundancy	5.8 (4.9)	13.1 (12.8)
**Refinement**		
Resolution (Å)	46.7-2.01	28.17-2.20
No. of reflections (refinement/ R_{free})	23910/2321	36635/3466
R / R_{free}	0.192/0.235	0.185/0.236
No. atoms		
Protein	2276	4936
Ligands	60	101
Water	155	164
R.m.s. deviations from ideal		
Bond lengths (Å)	0.008	0.02
Bond angles (°)	0.969	1.58
Ramachandran statistics (%)		
Favored	98.2	94.5
Allowed	1.8	5.0
Outliers	0	0.5
PDB code	6qkv	6qh8

*The highest resolution shell is shown in parentheses.

Table S2. Thermodynamic parameters of AdoHcy binding to YibK variants determined by ITC. The mean errors are derived from iterative fit of one-site binding model and estimated by unbiased Monte Carlo statistics using SEDPHAT.[1]

Proteins	Stoichiometry (n)	K_d (μM)	ΔH ($k$cal mol^{-1})	ΔS (cal $mol^{-1}K^{-1}$)	ΔG ($k$cal mol^{-1})
*l*YibK	0.55	8.80	-16.50	-32.6	-6.89
	±0.01	±0.01	±0.02	±0.12	±0.003
*c*YibK	0.69	8.93	-20.06	-44.2	-6.88
	±0.02	±0.02	±1.67	±0.32	±0.002

Table S3. Kinetic parameters of urea-induced unfolding of *l*YibK and *c*YibK.

	Kinetic phase	$k_f^{H_2O}$ (s^{-1})	m_f (kcal mol^{-1} M^{-1})	$k_u^{H_2O}$ (s^{-1})	m_u (kcal mol^{-1} M^{-1})	$D_{50\%,kin}$ (M)[a]	ΔG_{kin} (kcal mol^{-1})[a]
*l*YibK	Fast	65.8±14.6	-3.14±0.17	0.026±0.005	0.27±0.03	2.36±0.21	4.62±0.17
	Slow	6.42±1.28	-2.30±0.12	$(9.50\pm4.64)*10^{-6}$	1.33±0.08	3.70±0.21	7.94±0.31
*c*YibK	Fast	124±129	-2.28±0.45	0.385±0.063	~0[b]	N.D.[c]	N.D.[c]
	Slow	2.41±1.13	-1.71±0.13	$(6.55\pm3.60)*10^{-7}$	1.35±0.08	4.94±0.34	8.95±0.43

a. The transition points ($D_{50\%,kin}$) and free energies of unfolding (ΔG_{kin}) were derived from the kinetic parameters associated with the fast and slow phases.

b. The unfolding arm of the slow kinetic phase of *c*YibK showed no apparent denaturant concentration-dependency.

c. The change of unfolding kinetic rate as a function of urea concentration is too small to be accurately determined.

Supplementary Figures

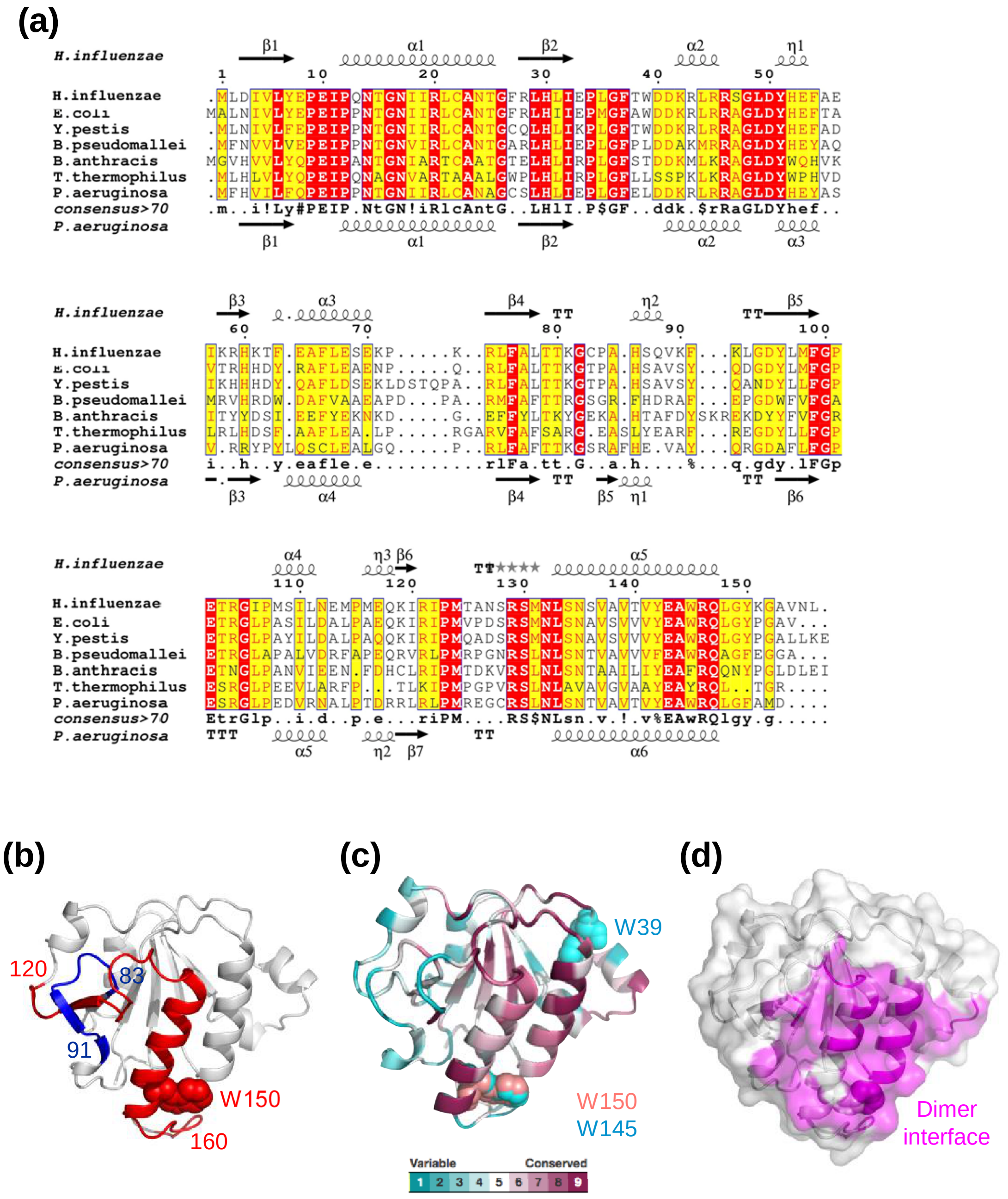

Figure S1. Sequential and structural conservation of YibK variants. (**a**) A sequence alignment of YibKs from different organisms. (**b**) Cartoon representation of *c*YibK with the knotting loop and threading C-terminal helix shown in blue and red, respectively. (**c**) Structural mapping of sequential conservation amongst YibK variants. Residues that are identical and similar in the sequence alignment are colored in red and salmon, respectively. The tryptophan side -chains in *Pa*YibK and *Hi*YibK are shown in salmon and cyan spheres, respectively, with the identities indicated. (**d**) Surface representation of monomeric YibK with the dimer interface colored in magenta.

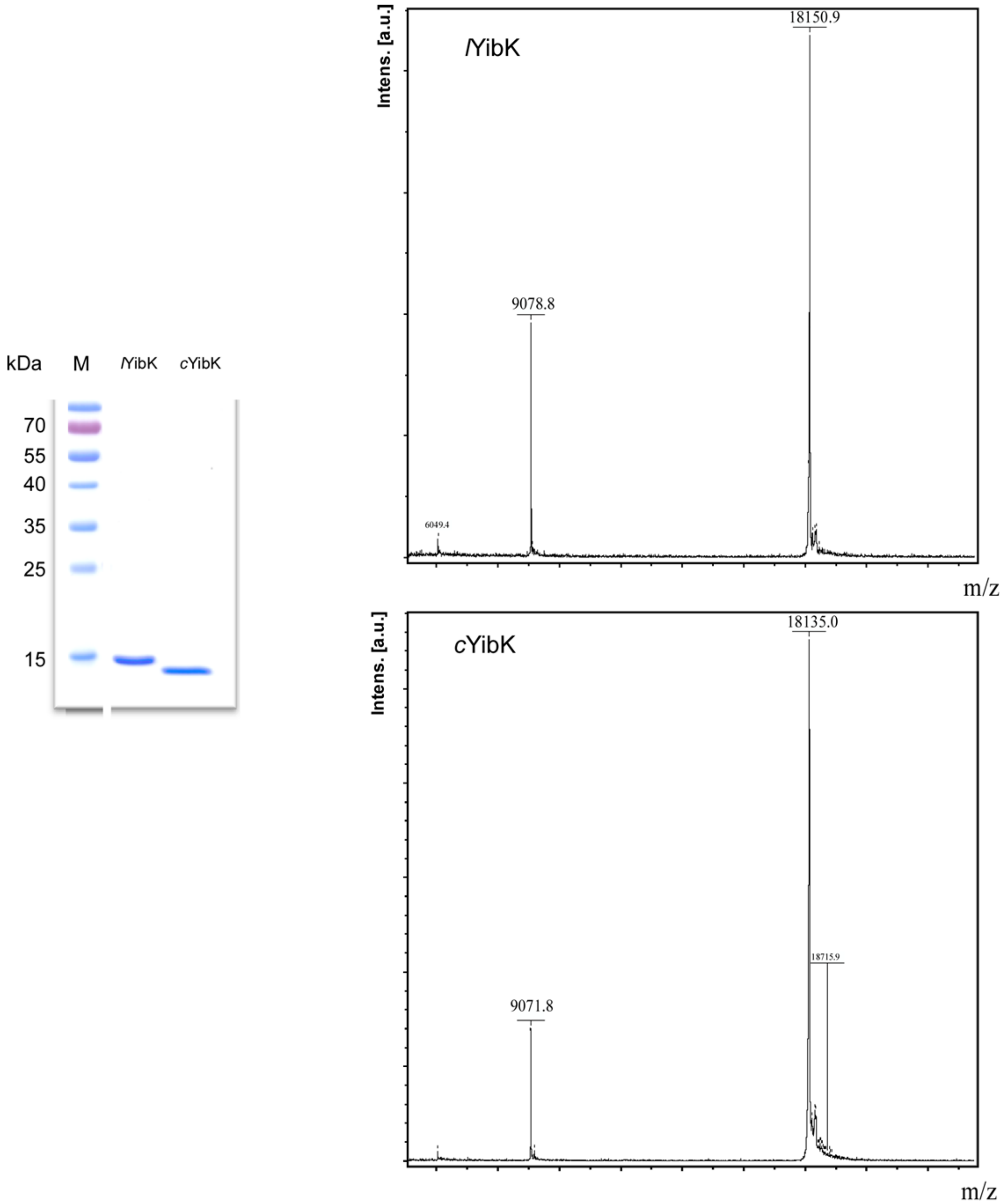

Figure S2: **SDS-PAGE analysis and Mass spectrometry.** Purified *c*YibK and *l*YibK (circular and linear forms of *Pa*YibK_lo) were analyzed by SDS-PAGE and MALDI-TOF mass spectrometry.

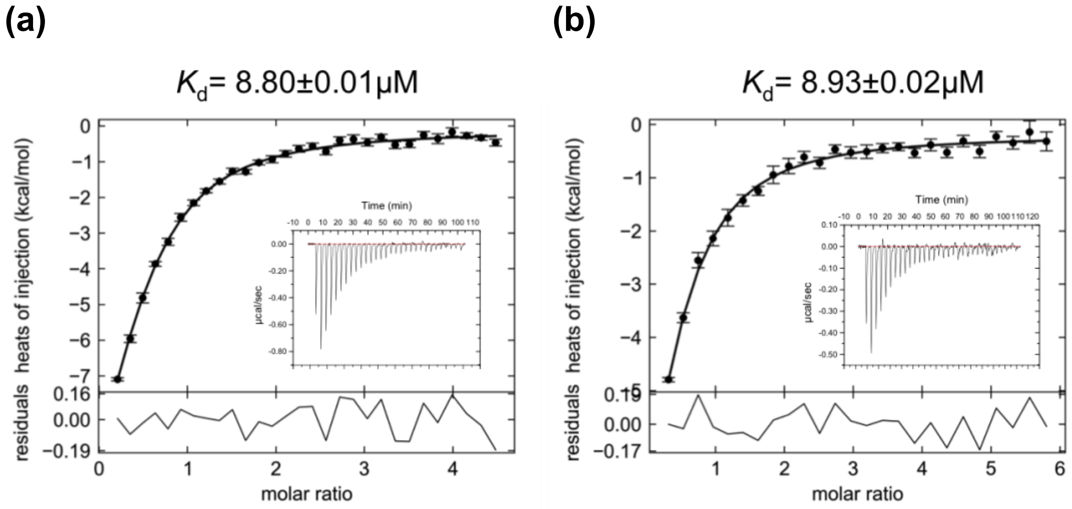

Figure S3. Isothermal titration calorimetry (ITC) thermograms of *l*YibK and *c*YibK. The titration heat for (**a**) *l*YibK and (**b**) *c*YibK were defined and integrated using integration algorithm of NITPIC.[3] Insets: the heat power of series of titration peaks as a function of the time duration. The thermodynamic parameters of cofactor AdoHcy binding were determined by fitting the integrated data to the one-site binding model. Fitting residues for each titration plot were shown in lower panel.

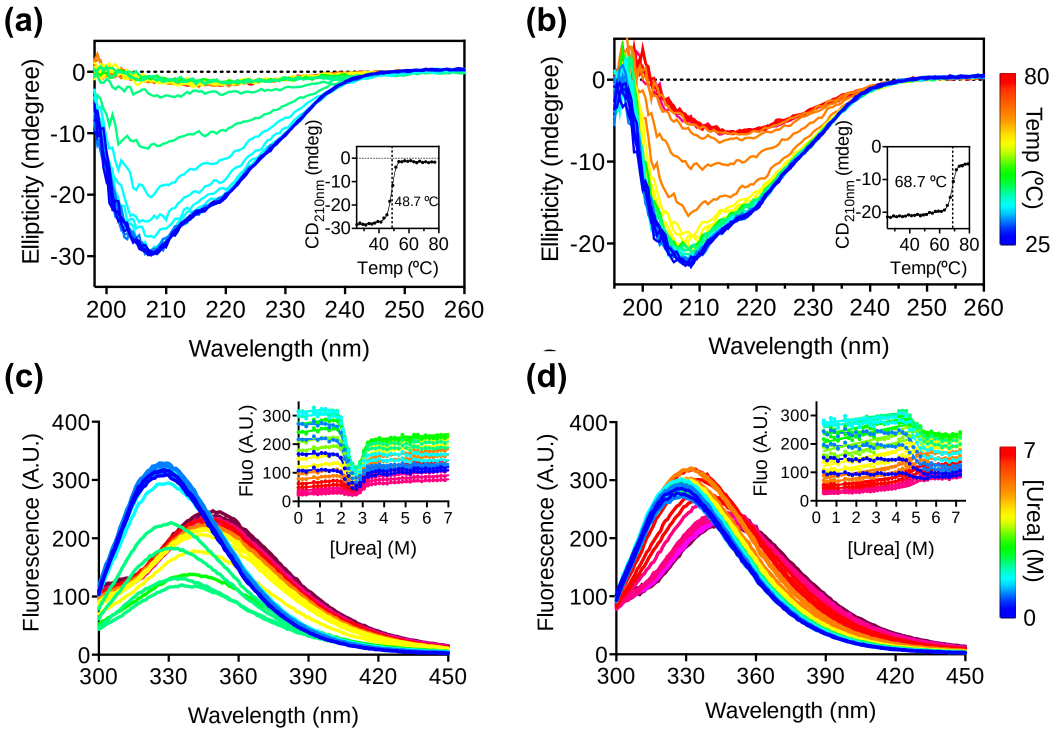

Figure S4. Equilibrium thermal and urea-induced denaturation of *l*YibK and *c*YibK. Equilibrium thermal denaturation of (**a**) *l*YibK and (**b**) *c*YibK monitored by the change of far-UV CD signal. Series of spectra of *l*YibK and *c*YibK were recorded from 25 to 80°C with a linear gradient with equal space 2°C, color-ramped from blue to red. The inset panels are the change of CD signal at 210 nm as a function of temperature. The transition temperatures were derived by fitting of two-state equilibrium model and indicated by the dash lines. Urea-induced equilibrium denaturation of (**c**) *l*YibK and (**d**) *c*YibK monitored by the change of intrinsic tryptophan fluorescence. Series of spectra of *l*YibK and *c*YibK were recorded from 0 to 7 M urea with a linear gradient with equal space, color-ramped from blue to red. The inset panels are the transition curves based on the fluorescence changes at different wavelengths as a function of urea concentration and the solid line is the nonlinear degeneration curves fit to the three state folding model as described previously.[4]

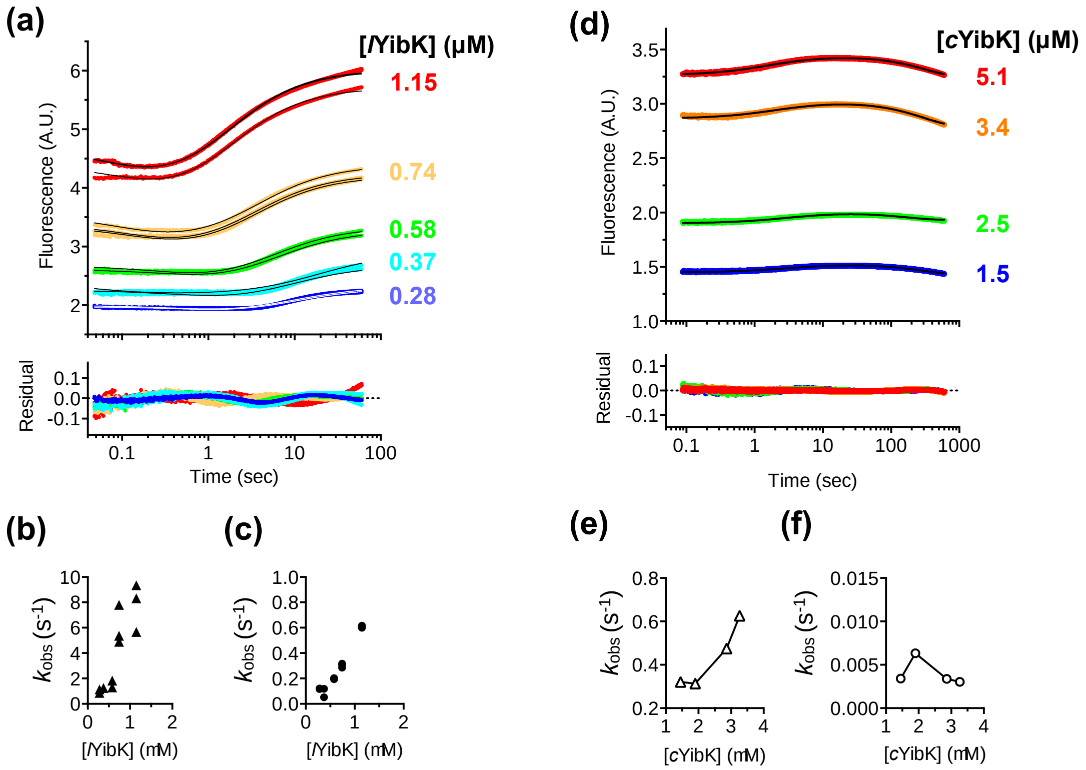

Figure S5. *l*YibK and *c*YibK refolding kinetic traces at different protein concentrations monitored by intrinsic fluorescence. (**a**) Refolding kinetic traces of *l*YibK at the different protein concentrations by removal of urea from 7.2 M to the final 0.65 M. The refolding kinetic rates were fit to the double second-order kinetic model as described previously.[7] The continuous solid lines represent the best fit of the data to the second-order kinetic model. The fitting residuals were shown in the lower panel. (**b**) The [*l*YibK] dependence of the observing rates (k_{obs}) of fast refolding phase. (**c**) The [*l*YibK] dependence of the observing rates (k_{obs}) of the slow refolding phase. (**d**) Refolding kinetic traces of *c*YibK at the different protein concentrations by removal of urea from 7.2 M to the final 3.38 M. The fast refolding kinetic rates were derived and fit to the second-order kinetic model. The slow rates were derived by nonlinear regression to a single exponential function. The continuous solid lines represent the best fit of the data to the kinetic models. The fitting residuals were shown in the lower panel. (**e**) The [*c*YibK] dependence of the observing rates (k_{obs}) of the fast refolding phase. (**f**) The [*c*YibK] dependence of the observing rates (k_{obs}) of the slow refolding phase.

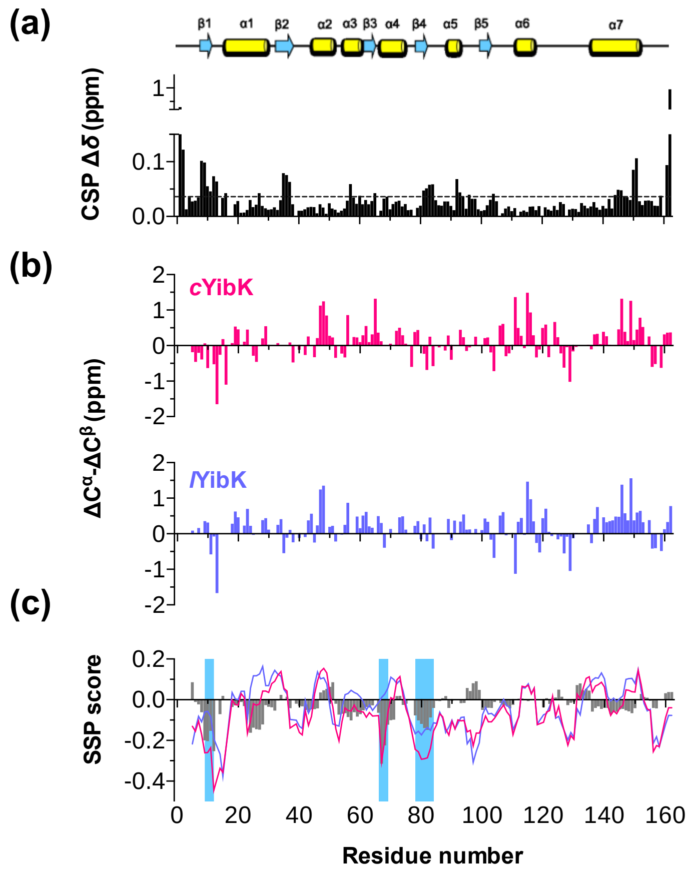

Figure S6. Chemical shift perturbations induced by backbone cyclization and comparative analyses of the secondary structure propensity of *l*YibK and *c*YibK. (**a**) The chemical perturbations (CSP) as a function of residue number of YibK. The dash line indicates the mean value of CSP. (**b**) The secondary chemical shifts of *l*YibK (orchid blue) and *c*YibK (magenta) were calculated using Cα and Cβ chemical shifts deviated from the random coil chemical shifts.[5] (**c**) The secondary structure propensity of *l*YibK (orchid blue line) and *c*YibK (magenta line) was evaluated using SSP software suite[6] and shown along with the difference plot $\Delta SSP_{cYibK\text{-}lYibK}$ (gray columns) between *c*YibK and *l*YibK. The increased secondary structure propensity regions were represented in the shaded areas colored in aqua blue.

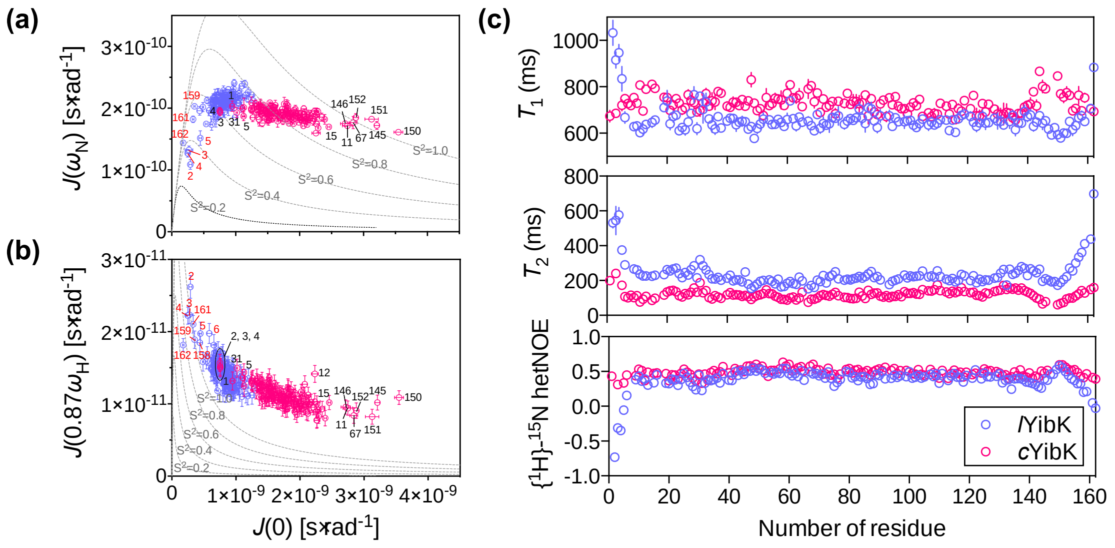

Figure S7. Reduced spectra density mapping of backbone dynamics of *l*YibK and *c*YibK derived from NMR T_1 and T_2 relaxation, and static $\{^1H\}$-^{15}N hetNOE measurements. Correlation plots of (**a**) $J(0)$ *versus* $J(\omega_N)$ and (**b**) $J(0)$ *versus* $J(\omega_H)$ reduced spectra densities of backbone dynamics of *l*YibK (orchid blue) and *c*YibK(magenta). The dash gray lines represents the theoretical values of the spectra density calculated at the pairs of frequencies, which assume isotropic rigid-body rotation with different order of parameters (S^2). (**c**) NMR T_1 and T_2 relaxation, and static $\{^1H\}$-^{15}N hetNOE measurements of *l*YibK (orchid blue) and *c*YibK (magenta).

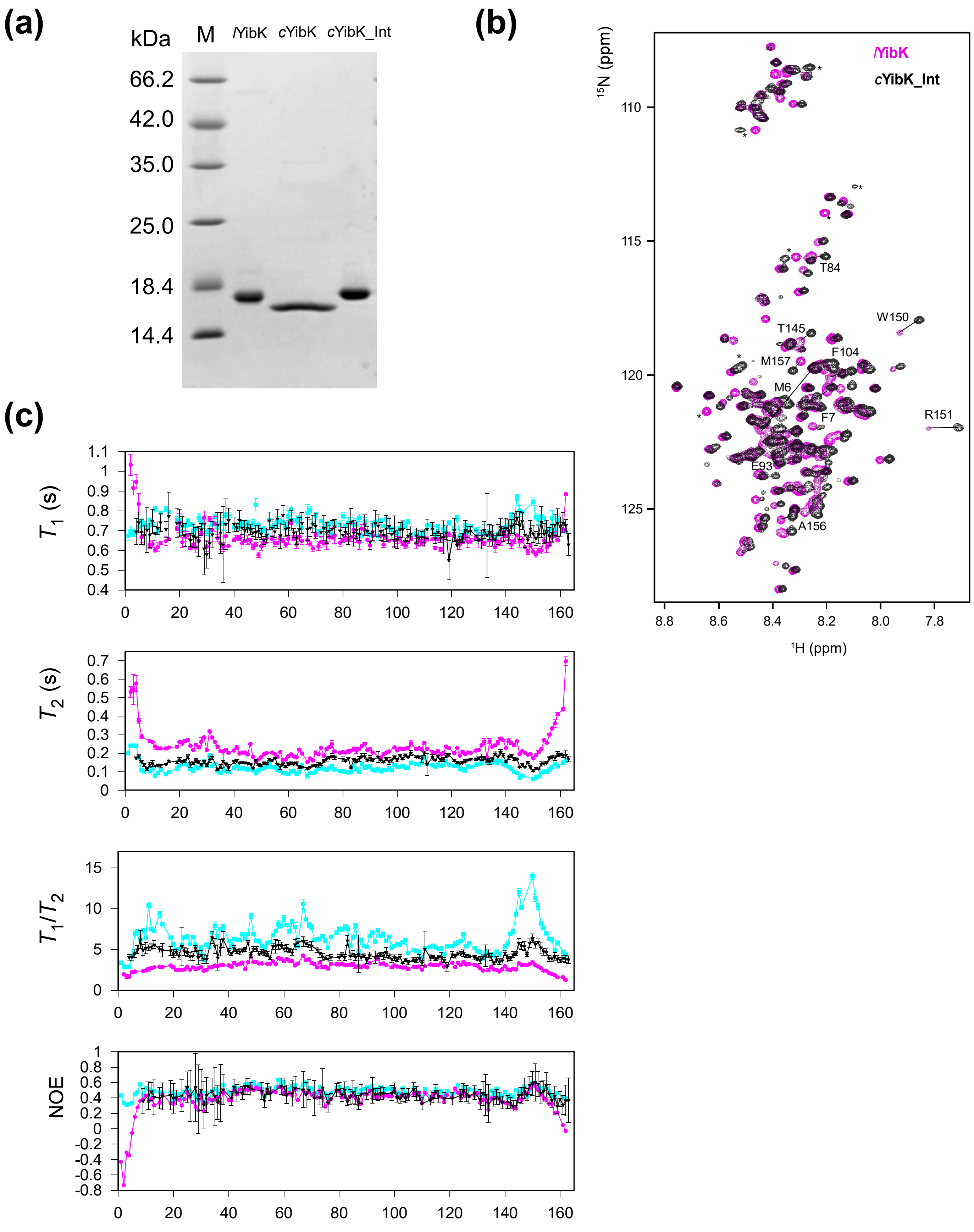

Figure S8: ***c*YibK_Int (*c*YibK without knotting)** (**a**) Comparison of *l*YibK, *c*YibK, and *c*YibK_Int by SDS-PAGE analysis. The presence of His-tag in *c*YibK_Int induced a mobility shift. (**b**) An overlay of [^{1}H,^{15}N]-HSQC spectrum of *c*YibK (magenta) and *c*YibK_Int (black). Asterisks indicate peaks with large shifts due to the differences in the primary structure. (**c**) Comparison of ^{15}N relaxation analysis of *c*YibK_Int (black), *c*YibK (cyan), and *l*YibK (magenta).

Supplementary references

(1) Houtman, J. C.; Brown, P. H.; Bowden, B.; Yamaguchi, H.; Appella, E.; Samelson, L. E.; Schuck, P. *Protein Sci* **2007**, *16*, 30.
(2) Robert, X.; Gouet, P. *Nucleic Acids Res* **2014**, *42*, W320.
(3) Keller, S.; Vargas, C.; Zhao, H.; Piszczek, G.; Brautigam, C. A.; Schuck, P. *Anal Chem* **2012**, *84*, 5066.
(4) Lou, S. C.; Wetzel, S.; Zhang, H.; Crone, E. W.; Lee, Y. T.; Jackson, S. E.; Hsu, S. T. *J Mol Biol* **2016**, *428*, 2507.
(5) De Simone, A.; Cavalli, A.; Hsu, S. T. D.; Vranken, W.; Vendruscolo, M. *J Am Chem Soc* **2009**, *131*, 16332.
(6) Marsh, J. A.; Singh, V. K.; Jia, Z. C.; Forman-Kay, J. D. *Protein Sci* **2006**, *15*, 2795.
(7) Mallam, A. L.; Jackson, S. E. *J Mol Biol* **2006**, *359*, 1420.
